# How Does It Look in an Autopsy? Hepatosplenic T-Cell Lymphoma in a Patient with Crohn’s Disease on Azathioprine

**DOI:** 10.4274/tjh.galenos.2020.2020.0077

**Published:** 2020-08-28

**Authors:** Pulkit Rastogi, Ritambhra Nada

**Affiliations:** 1 Postgraduate Institute of Medical Education and Research, Department of Histopathology, Chandigarh, India

**Keywords:** Crohn’s disease, Hepatosplenic T-cell lymphoma, Azathioprine, Autopsy

A 29-year-old male known to have refractory Crohn’s disease and taking azathioprine for 18 months developed fever, jaundice, and hepatosplenomegaly for 15 days. Complete blood counts revealed pancytopenia (hemoglobin: 82 g/L; white blood cell count: 2.6x10^9^/L; platelet count: 21x10^9^/L; no abnormal cells in peripheral blood). He suffered cardiac arrest and an autopsy was conducted. The small intestine revealed skip lesions with deep submucosal ulcers and intervening normal areas (Figure 1A). The liver was enlarged with prominent intrasinusoidal, portal, and perivenular infiltrate of small to intermediate sized atypical lymphoid cells, which were CD3^pos^/CD2^pos^/CD5^pos^/CD7^pos^/CD4^neg^/CD8^pos^/grazymeB^neg^/CD30^neg^/CD20^neg^/PAX5^neg^/CD56^neg^ (Figures 1B, 1C, 1D). This infiltrate was seen in the splenic red pulp, glomeruli, renal interstitium, sinusoids of bone marrow, major branches of the pulmonary artery (leading to lung infarcts), and alveolar septae (Figures 1E, 1F, 1G, 1H). Fluorescence in situ hybridization (Figure 1I; three red marks, 7q31 red probe) revealed isochromosome 7q [i(7q)]. Overall findings confirmed hepatosplenic T-cell lymphoma (HSTL).

Ten percent of cases of HSTL develop in inflammatory bowel disease patients on thiopurine [1]. A multistep process resulting in selection and malignant transformation of γδ-T-cell clones in Crohn’s disease is believed to be the underlying event [2]. The presented case emphasizes the relevance of medical autopsy in such puzzling cases by providing the complete pathology spectrum of the disease for understanding its pathobiology.

## Figures and Tables

**Figure 1:**
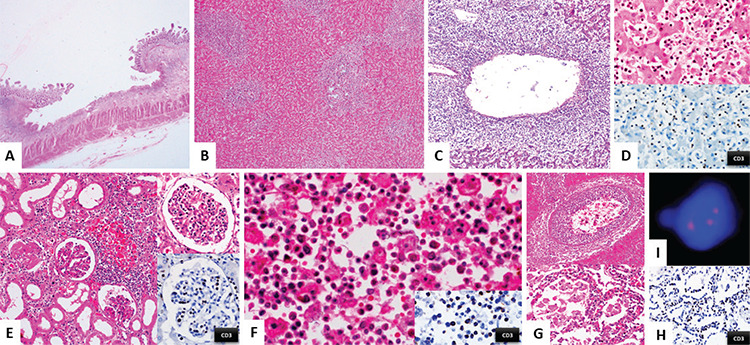
A) Small intestine with deep submucosal ulcers. B, C, D) Liver with a prominent portal and intrasinusoidal and perivenular infiltrate of small to intermediate sized atypical lymphoid cells that are CD3pos. This infiltrate was seen in glomeruli and the renal interstitium (E), sinusoids of bone marrow (F), major branches of the pulmonary artery (G), and alveolar septae (G, H). I) Fluorescence in situ hybridization (three red signals: 7q31 red probe) revealed isochromosome 7q [i(7q)].
